# Impact of label-free technologies in head and neck cancer circulating tumour cells

**DOI:** 10.18632/oncotarget.12086

**Published:** 2016-09-16

**Authors:** Arutha Kulasinghe, Liz Kenny, Chris Perry, Jean-Paul Thiery, Lidija Jovanovic, Ian Vela, Colleen Nelson, Chamindie Punyadeera

**Affiliations:** ^1^ The School of Biomedical Sciences, Institute of Health and Biomedical Innovation, Queensland University of Technology, Kelvin Grove, Queensland, Australia; ^2^ School of Medicine, University of Queensland, Royal Brisbane and Women's Hospital, Herston, Queensland, Australia; ^3^ Department of Otolaryngology, Princess Alexandra Hospital, Woolloongabba, Queensland, Australia; ^4^ Department of Biochemistry, Yong Loo Lin School of Medicine, National University of Singapore, Singapore; ^5^ Australian Prostate Cancer Research Centre-Queensland, Institute of Health and Biomedical Innovation, Queensland University of Technology, Brisbane, Australia; ^6^ Department of Urology, Princess Alexandra Hospital, Wolloongabba, Queensland, Australia

**Keywords:** circulating tumour cells, head and neck cancers, label free capture, metastasis, CellSearch^®^

## Abstract

**Background:**

The ability to identify high risk head and neck cancer (HNC) patients with disseminated disease prior to presenting with clinically detectable metastases holds remarkable potential. A fraction of circulating tumour cells (CTCs) are invasive cancer cells which mediate metastasis by intravasation, survival and extravasation from the blood stream to metastatic sites. CTCs have been cleared by the FDA for use as surrogate markers of overall survival and progression free survival for breast, prostate and colorectal cancers using the CellSearch^®^ system. However, the clinical significance of CTCs in head and neck cancer patients has yet to be determined. There has been a significant shift in CTC enrichment platforms, away from exclusively single marker selection, to epitope-independent systems.

**Methods:**

The aim of this study was to screen advanced stage HNC patients by the CellSearch^®^ platform and utilise two other epitope-independent approaches, ScreenCell^®^ (microfiltration device) and RosetteSep^™^ (negative enrichment), to determine how a shift to such methodologies would enable CTC enrichment and detection.

**Results:**

In advanced stage HNC patients, single CTCs were detected in 8/43 (18.6%) on CellSearch^®^, 13/28 (46.4%) on ScreenCell^®^ and 16/25 (64.0%) by RosetteSep™ (the latter could also detect CTC clusters). Notably, in patients with suspicious lung nodules, too small to biopsy, CTCs were found upon presentation. Moreover, CTCs were readily detected in advanced stage HNC patients.

**Conclusion:**

The epitope-independent platforms detected higher CTC numbers and clusters. Further studies are needed to ascertain whether CTCs can be used as independent prognostic markers for HNCs.

## INTRODUCTION

Head and neck squamous cell carcinomas (HNSCCs) account for the sixth commonest cause of cancer mortality worldwide with an incidence of 650,000 cases annually [1, 2]. The combined 5 year survival rate is 50%, with poor control of distant metastatic spread [2]. Approximately 50-60% of HNSCC patients develop locoregional recurrence of which a further 20% progress to distant metastasis (DM), accounting for an increasing rate of treatment failure [3]. Human Papillomavirus (in particular HPV-16,-18) has been etiologically linked with HNCs and has a better general prognosis at presentation compared to HPV-negative HNCs [4–6]. In fact, patients with HPV+ oropharyngeal cancers have better outcomes than their HPV-negative counterparts at similar advanced stages of disease [5]. Five year survival rates for this same cohort is much better than the HPV-negative patients [7]. Whilst DM rates in HPV+ and HPV- patients appears to be quite similar [8, 9], HPV+ DM tends to occur after longer time periods, in unusual and multiple sites (lungs, brain, kidney, skin, and skeletal muscle) [4, 10, 11]. This may have clinical ramifications with the requirement for longer clinical follow-ups and monitoring in HPV-positive patients.

The dissemination of cancer from the primary tumour to lymph nodes and distant organs, such as the lungs, liver and bone portends poorer patient prognosis [12]. The spread of cancer cells can occur through invasion of local tissue, lymphatics, and/or through the blood circulation as CTCs [12, 13]. Moreover, CTCs can also be derived from metastatic deposits [14]. CTCs can be found in the blood as single cells and/or cell clusters, which can vary in their degree of epithelial and mesenchymal characteristics [14–16]. CTCs are believed to later extravasate from the blood and colonize distant organs, forming clinically detectable metastases [14]. The detection of CTCs in blood samples, coined as, ‘liquid biopsy’ is considered as a surrogate marker for patient response to treatment, thereby directing therapeutic decisions [14, 17, 18].

Current diagnostic methods for staging include clinical assessment, sophisticated imaging and often biopsy of the primary site as well as suspected metastasis. When metastases are clinically evident, treatment is palliative. There is a need for better prognostic markers to determine patients who are at an increased risk of locoregional recurrence and/or distant metastatic disease [1, 19–21]. CTC numbers detected in the blood have shown correlation with tumour staging and advanced disease in breast, prostate, colorectal and lung cancers [2, 22]. More recently, the isolation, culture and drug sensitivity of CTCs has also come to the fore opening up avenues for targeted treatment strategies and personalized medicine [23–26]. Limited CTC studies in patients with HNC were carried out in small patient cohorts. There is also a lack of standardized methodologies for CTC enumeration in HNC and it remains clinically unvalidated [1, 2, 12, 19, 27, 28].

We hypothesize that the enumeration of CTC from HNSCC patients can be increased by using epitope-independent detection platforms. The aim of this study was to evaluate (i) CellSearch^®^ (FDA-approved CTC platform) and (ii) epitope-independent technologies: ScreenCell^®^ (microfiltration) and RosetteSep™ (negative enrichment) in patients with locally advanced HNSCC to determine the CTC capture efficiency of such assays. The ScreenCell^®^ and RosetteSep™ assays were followed up by immunocytochemistry for the detection of CTCs. EGFR expression was incorporated into the CTC panel as it has shown to be detectable on the cell surface of HNSCC CTCs [29, 30]. To our knowledge, this is the largest paired patient study conducted across multiple CTC detection platforms in HNC patients. Our data reveals that the epitope-independent platforms detect higher single CTC numbers as well as CTC clusters. Larger clinical studies are warranted to ascertain whether CTCs can be used as independent prognostic markers for HNCs.

## RESULTS

### Patient characteristics

A total of 58 patients were included in this study. Patient characteristics are shown in Table 1. The median age was 60 years (range 40-87); men (*n* = 50), women (*n* = 8). The most distinctive histology was mucosal oral cavity SCC (68.1%) followed by oropharyngeal (22.4%), nasopharyngeal (6.9%) and laryngeal SCC (8.6%). The bulk of patients presented with tumour grade ranging from T4 (55.1%) to advanced nodal spread (N2A-C) (62.1%). The HPV-status was determined in all patients (*n* = 58) and found to be positive for HPV-16 (*n* = 14). DM was assessed by a multidisciplinary team (MDT) at the PAH by combining CT/PET-CT and MRI scans. 98.3% of the patients were found to be radiographically M0. CTC presence was determined from new patients upon presentation to the clinic, prior to therapy. Upon patient follow up, two patients in the study had succumbed to their disease. One patient (T4N2CM0 oropharyngeal SCC, HPV-16 positive) treated with chemotherapy (Docetaxel, cisplatin and 5-fluorouracil) died within 11 days and the other patient (T4N2BM0 supraglottic SCC) who had mildly avid FDG-PET uptake on the in the right apex of the lung died in 24 days (See Figure 4). Notably, both patients presented with CTC clusters in their blood post RosetteSep™ enrichment.

**Table 1:** Patient demographic (*n*=58) including Age, Gender, Tumour Site, Nodal Status, Distant Metastases, HPV Status

Variables	*N*
Total	58 (100%)
Gender	
Male	50 (86.2%)
Female	8 (13.8%)
Age, y	
<60	19 (32.8%)
>60	39 (67.2%)
***Anatomic site of primary***	
Oral Cavity	36 (68.1%)
Oropharyngeal	13 (22.4%)
Nasopharyngeal	4 (6.9%)
Laryngeal	5 (8.6%)
***Tumour Size***	
T1	2 (3.4%)
T2	4 (6.9%)
T3	9 (15.5%)
T4	32 (55.1%)
Tx	11 (19.0%)
***Nodal spread***	
N0	21 (36.2%)
N1	1 (1.7%)
N2a-c	36 (62.1%)
***Distant metastases***	
M0	57 (98.3%)
M1	1 (1.7%)
***HPV status***	
HPV-positive	14 (24.1%)
HPV-negative	44 (75.9%)

### Spike in experiments were performed to serve as positive controls for the three platforms

When FaDu cells (EpCAM+ cells) of clinically relevant numbers (<100 cells) were spiked into blood (10ml) and recovery studies performed, CellSearch^®^ consistently detected over 95% of the cells, ScreenCell^®^ 60% and RosetteSep™ 70%. It should be noted that as the detection of tumour cells on the CellSearch^®^ is fully automated whereas the epitope-independent techniques are manually processed post enrichment which including multiple wash steps.

### CellSearch^®^

**Figure 1:**
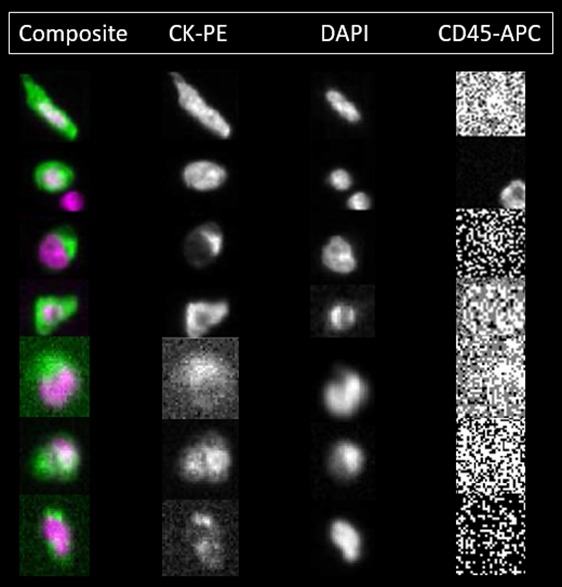
Patient CTCs detected by the CellSearch system The second row shows a nucleated, CD45 positive cell below the CTC which is a leukocyte below the patient CTC. The varying morphology of patient CTCs is evident from Figure 1 where some CTCs appear epithelial like in morphology whereas some appear fibroblast-like and mesenchymal. Imaged on the Celltracks Analyzer II ^®^ system.

The CellSearch^®^ system was able to successfully identify CTCs in 8/43 (18.6%) patients with locally advanced HNSCC before therapy (Table 2 and Figure 1). The number of CTCs detected per 7.5 mL of blood ranged from 0 to 28 (0 CTC: *n* = 35 patients; 1 CTC: *n* = 5 patients; 2 CTCs: *n* = 2 patients, 28 CTCs: *n* = 1 patient). All 8 CTC-positive patients detected by the CellSearch^®^ were HPV-16 negative. CTCs were not detected in any of the bloods analysed from normal healthy volunteers (NHV) controls (*n* = 4). There was no significant correlation between the presence of CTCs (*p* = 0.73; Pearson's chi-squared test) and tumour staging. In the patient presenting with 28 CTCs/7.5ml whole blood, the patient had extensive nodal disease and suspicious lung nodules.

**Table 2:** Correlation between tumour stage and CTC presence across the CellSearch^®^ ScreenCell^®^ and RosetteSep™ platforms

CellSearch (n=43					ScreenCell (n=28)					RosetteSep (n=25)			
													
Variables	CTC positive	CTC negative	P		Variables	CTC positive	CTC negative	P		Variables	CTC positive	CTC negative	P
													
Total (n=43)	8/43 (18.6%)	35/43 (81.4%)	0.73		Total (n=28)	13/28 (46.4%)	15/28 (53.6%)	0.04*		RosetteSep (n=25)	16/25 (64.0%)	9/25 (36.0%)	
T1 (n=2)	0	2			T1 (n=0)	0	0			T1 (n=0)	0	0	0.918
T2-T4 (n=34)	7	27			T2-T4 (n=22)	8	14			T2-T4 (n=22)	14	8	
TX (n=7)	1	6			TX (n=4)	5	1			TX (n=3)	2	1	
													
N0 (n=8)	1	7	0.77		N0 (n=6)	3	3	0.52		N0 (n=4)	4	0	0.10
N1 (n=1)	0	1			N1 (n=1)	1	0			N1 (n=0)	0	0	
N2a-c (n=34)	7	27			N2a-c (n=21)	9	12			N2a-c (n=21)	12	9	
													
M0 (n=39)	7	32	0.41		M0 (n=25)	11	14	0.54		M0 (n=1)	0	1	0.17
M1 (n=2)	0	2			M1 (n=1)	1	0			M1 (n=0)	0	0	
MX (n=2)	1	1			MX (n=2)	1	1			MX (n=24)	16	8	
													
Stage I (n=0)	0	0	0.16		Stage I (n=0)	0	0	0.78		Stage I (n=0)	0	0	0.39
Stage II (n=1)	0	1			Stage II (n=0)	0	0			Stage II (n=0)	0	0	
Stage III (n=4)	0	4			Stage III (n=4)	2	2			Stage III (n=0)	0	0	
Stage IVA (n=37)	8	29			Stage IVA (n=22)	10	12			Stage IVA (n=21)	15	6	
Stage IVC (n=1)	1	0			Stage IVC (n=3)	2	1			Stage IVC (n=4)	2	2	

CTC positive: Cytokeratin (8, 18, 19) positive, negative for CD45, larger than leukocytes with intact nuclei. CTC negative: Negative for cytokeratin (8,18,19), positive for CD45.

### ScreenCell^®^

**Figure 2:**
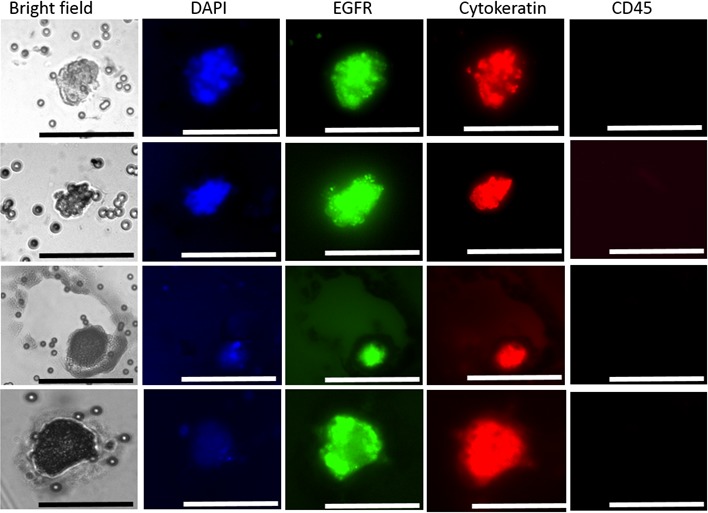
Presentation of selected CTCs in the peripheral blood of HNC patients Image gallery presenting CTCs isolated by the ScreenCell^®^ microfiltration system: Cytokeratin, EGFR, DAPI-positive but CD45 negative with a cell size larger than the pore size of the filter (approximately 7μm). Imaged on the Olympus IX3 inverted microscope. Scale bar represents 50μm.

The ScreenCell^®^ platform was able to successfully capture CTCs on the filter membrane in 13/28 (46.4%) patients with locally advanced HNSCC before therapy (Table 2, Figure 2). The number of single CTCs detected per 3 mL of blood ranged from 0 to 3 (0 CTC: *n* = 14 patients; 1 CTC: *n* = 3 patients; 2 CTCs: *n* = 6 patients; 3 CTCs: *n* = 1 patient) and CTC clusters (5 or more cells) *n* = 5 patients. In two CTC-positive patients detected by ScreenCell^®^, there was concurrent HPV-16 positivity and both patients had base-of-tongue (BOT) SCC and were stage T2-4, N2b. CTCs were not detected in NHV control samples (*n* = 4). There was a significant correlation (*p* = 0.04; Pearson's chi-squared test) found between the presence of CTCs before therapy and the patient tumour staging.

### RosetteSep™

**Figure 3:**
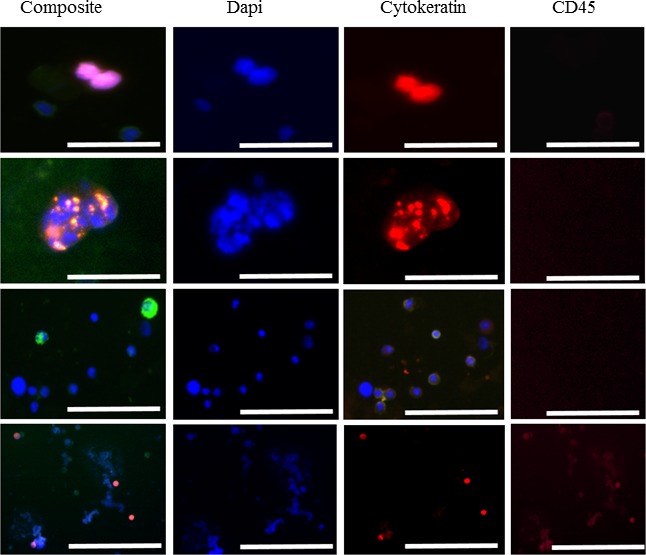
Presentation of selected CTCs in the peripheral blood of HNC patients Image gallery presenting CTCs captured by the RosetteSep™ system: Cytokeratin, EGFR, DAPI-positive but CD45 negative. Imaged on the Olympus IX3 inverted microscope. Scale bars represent 50 μm.

RosetteSep™ was able to successfully negatively deplete whole blood and enrich for CTCs in 16/25 (64.0%) patients with locally advanced HNSCC before therapy (Table 2, Figure 3). The number of single CTCs ranged from 0 to 3, (0 CTC: *n* = 9 patients; 1 CTC: *n* = 6 patients; 2 CTCs: *n* = 5 patients; 3 CTCs: *n* = 1 patients), CTC doublets (2 CTCs) and CTC clusters (CTC doublets: *n* = 1 patients; CTC clusters: *n* = 4 patients). A few patients had concurrent single CTCs and CTC clusters detectable post enrichment. Notably, in one of the patients who died during the study period, RosetteSep™ was able to successfully isolate CTC clusters from only 3ml of whole blood (Figure 3). 6/25 (24%) of the HNSCC patients were HPV-16 positive with 4/6 (66.7%) presenting with single CTCs and CTC clusters. CTCs were not detected in 8 NHV control samples. There was no significant correlation between the presence of CTCs and tumour staging (*p* = 0.918299; Pearson's chi-squared test).

### Head to head comparison between platforms

There was concordance between the CellSearch^®^ and ScreenCell^®^ platforms in detecting CTCs across paired bloods from the same patient for 2 patients (tumour stage T4, nodal spread N2b) (Supplementary Figure 6). However, four further patients (tumour stage T3-4, N2b-c) showed detectable CTCs on the CellSearch^®^ only and 11 patients (tumour stage T2-T4, N0-N2b) showed CTCs on the ScreenCell^®^ only, including CTC clusters.

When ten paired advanced stage HNSCC patient bloods were enriched using the CellSearch^®^ and RosetteSep™ platforms, CTCs were detected in 1/10 by CellSearch^®^ and 6/10 by RosetteSep. In the case where the CellSearch^®^ detected 1 CTC, it was similarly detectable post RosetteSep™ enrichment (T3N2b supraglottic SCC). The RosetteSep™ platform was able to detect CTC clusters more readily and with higher detection rates in the analysed patient cohort.

In all patients presenting with CTC clusters (*n* = 4), outcomes were poor. Two patients died in less than a month and the other two patients were referred onto palliative care due to worsening outcomes.

## DISCUSSION

This is the first study to compare CTC detection using three CTC enrichment platforms for HNCs. In our study, CTCs were detected using CellSearch^®^ (18.6%), ScreenCell^®^ (46.4%) and RosetteSep™ (64.0%) respectively. In the head to head comparisons, ScreenCell^®^ was able to capture more CTCs (single, clusters) than the CellSearch^®^ across 26 HNC patient samples. This might be due to a low or lack of EpCAM expression on CTCs of these patients possibly linked to engagement into EMT, whereby CellSearch^®^ would not detect these CTCs [1, 31, 32]. More importantly, the ScreenCell^®^ platform only processes 3ml of blood per sample compared to the 7.5 mL used in CellSearch^®^, therefore, in less than half the volume of blood, ScreenCell^®^ is able to isolate tumour cells more readily than the gold standard, CellSearch^®^ technology. This higher CTC capture efficiency has been highlighted in several studies [33–35]. The CTCs detected by CellSearch^®^ only could be ‘small CTCs’ which have been known to be found in circulation [31], which could be able to pass through the ScreenCell^®^ Filters and thereby remain undetectable. Importantly, the patient prognosis was found to be poorer when CTCs were detected (irrespective of enrichment platform), more so when CTC clusters were present in circulation.

In previous HNSCC studies, the CellSearch^®^ system was able to detect CTCs in 8/49 (16%) and 3/9 HNSCC patients [2, 19]. Buglione *et al* reported higher CTC numbers were found in stage IV than in stage I-III HNSCC patients [36]. He *et al* found that CTCs were more frequently detected in patients with nodal stage 2 and 3 than with nodal stage 0 or 1 [2]. In the study by Nichols *et al*., CTCs were detected in 6/15 (40%) of HNSCC patient who were queried with lung nodules [37]. In our study, CTCs were more readily detectable across all three platforms in tumour stages T2-T4 and nodal stages N2A-C, highlighting a higher volume of disease, consistent with previously published literature. It has been shown that in metastatic cancers, 20-50% of patients are negative for CTC in blood samples of 10ml and below. Therefore, higher blood volumes, with more frequent intervals may be required to properly access the burden of disease in HNC patients [31].

When paired blood samples from HNSCC patients were compared between the CellSearch^®^ and RosetteSep™ (*n* = 10), RosetteSep™ processing and subsequent staining was able to detect CTCs more frequently than CellSearch^®^ (60% vs 10% respectively). More importantly, RosetteSep™ was able to enrich for CTC clusters, which are proposed to have a higher metastatic potential and intravasate into blood via leaky blood vessels or at site of vascular mimicry [12, 15, 32]. It should be noted that with the post enrichment cytospins for RosetteSep™, there is an inherent cell loss and morphologic changes during the spinning steps and subsequent washes [38], which could translate to higher actual patient CTC counts post enrichment. CTC clusters and/or circulating tumour microemboli (CTMs) have been shown to be 23 to 50 times more likely to establish distant metastases than single CTCs [15]. These CTC clusters were found to be more resistant than single cells in circulation and cleared faster from circulation to distant sites [15].

From spiking experiments, CellSearch^®^ was the platform most consistently able to recover over 95% of tumour cells when compared to the ScreenCell^®^ and RosetteSep™. This is likely due to the automation of the CellSearch^®^ technology and the increased wash steps and handling steps post enrichment with the epitope-independent techniques. ScreenCell^®^ is further limited with a total blood volume of only 3ml. Furthermore, we could show that FaDu cells are functional post RosetteSep™ enrichment whereas with CellSearch^®^, the CTCs can only be enumerated. The RosetteSep™ process maintained cells in a proliferative and viable state. This is important for any downstream CTC cultures, which have shown potential across multiple tumour types including the creation of CTC cell lines [14, 23]

It is important to note that, CellSearch^®^ showed clinical relevance in the CTCs detected by the use of the EpCAM based-platform and threshold values were generated for metastatic breast (mBC), prostate (mPC), colorectal (mCRC) where (0-4 CTCs) for mBC, mPC, and (0-2 CTCs) for mCRC had favourable patient outcomes whereas (≥5 CTCs) for mBC, mPC and (≥3 CTCs) for mCRC had unfavourable patient outcomes. These large cross-institutional studies have not been done for HNCs. Moreover, the clinical significance of the CTCs captured by the epitope-independent technologies needs to be analysed to determine whether an increased capture of CTCs in circulation are actually of clinically relevant CTCs [39].

## CONCLUSIONS

There has been a significant shift in the CTC enrichment field where epitope-independent CTC isolation has been shown to capture a greater population of the CTCs in the circulation in an unbiased fashion. Moreover, recent technological improvements in the field of microfluidics has given rise to fast and efficient CTC enrichment [14, 40, 41]. This study highlights the shift in the field from marker based technologies (EpCAM-CellSearch^®^), to marker-independent isolation. If the presence of CTCs predicts for the development of clinically evident metastases, identifying these before metastases are clinically evident may allow for escalation of systemic treatment. With the improvements in isolation technologies and higher CTC capture efficiencies, it potentially allows *ex-vivo* CTC propagation [14, 23]. In patients with established metastatic disease, culturing CTCs will allow for the testing of drug sensitivities, which in turn may help guide the choice of systemic palliative chemotherapy and reduce the morbidity from ineffective treatment. More studies are needed in the HNC field, to investigate the character of the circulating tumour cells, determine whether HPV-infection plays a pivotal role, and provide threshold CTC counts for HNSCC patients in order to determine patient prognosis.

## MATERIALS AND METHODS

### Patient cohort

Ethics approval was obtained by the Metro South Health Service District Human Research Ethics Committee in accordance with the NHMRC's guidelines (HREC/12/QPAH/381) to collect blood from the Princess Alexandra Hospital (PAH). This study has UQ (2009000779) and QUT (1400000617) ethics approval. After written informed consent was obtained from the participants, blood samples were obtained from patients. Advanced stage treatment-naïve HNSCC patients were identified as tumour stage T3/4 with locoregional nodal spread. A 10 ml of blood was collected either at the clinic or prior to surgery into CellSave (Immunicon Inc., Huntingdon Valley, USA) preservative tube for processing on the CellSearch^®^. A 10 ml BD Vacutainer K2E tube (EDTA) (BD-Plymouth, UK) for processing on the ScreenCell^®^ and 10ml Heparin tube (BD-Plymouth, UK) for processing with RosetteSep™. The CellSave tube was stored at room temperature and processed within 72 hours and the BD Vacutainer EDTA and Heparin tubes processed within 4 hours. CTC detection was performed using CellSearch^®^, ScreenCell^®^ and RosetteSep™ platforms (from November 2013 until November 2015). Gender, age, race and ethnicity, smoking status, alcohol consumption, tumour staging, nodal spread, distant metastases assessment by radiologist and HPV status were collated per HNSCC patient. The comparative schematic is presented in Supplementary Figure 1.

### Cell culture

All cell lines used in these experiments were obtained from the ATCC (Manassas, VA). The following HNSCC cell lines were selected: FaDu (ATCC^®^HTB43^TM^) and CAL27 (ATCC^®^CRL2095^TM^) (Passage range 6-10). Cells were cultured under standard conditions in humidified incubators at 37°C, 5% CO_2_. To avoid the influence of culture media, all culture media conditions were standardized to RPMI-1640-Glutamax (Life Technologies, Inc) supplemented with 10% foetal bovine serum (Life Technologies, Inc) and 1% Penicillin-Streptomycin (Life Technologies, Inc).

**Figure 4:**
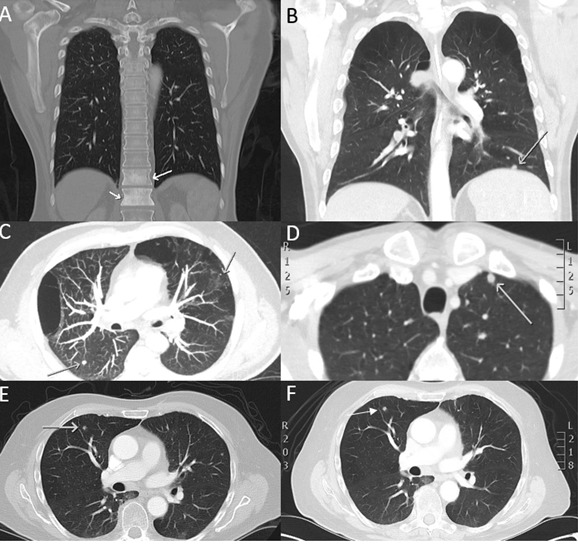
CT scan images of selected HNC patients presenting with suspicious HNC metastasis Bloods were taken upon presentation of these patients to clinic and CTCs assessed. Patient presenting with **A.** bony metastasis **B.**, **C.**, **D.** multiple suspicious lung metastasis **E.**, **F.** lung nodule which increased in size over 37 days.

### Enrichment and detection of circulating tumour cells using CellSearch^®^ (Veridex, NJ, USA)

Whole blood (7.5 ml) from CellSave Blood collection tubes (Veridex, NJ, USA) was mixed with 6.5ml of CellTracks^TM^ system buffer and centrifuged at 800 x g for 10 minutes and then placed on the AutoPrep^TM^ system (CellSearch^®^ Circulating tumour cell kit). The AutoPrep system added the appropriate reagents and magnetically separated the cells. The reagents include ferrofluid particles coated with EpCAM antibodies (positive selection) and the characterization antibodies (anti-CD45) conjugated to phycoerythrin (PE) and DAPI (nuclear stain). The cells were then quantitatively analysed with the CellTracks Analyzer. EpCAM-positive, cytokeratin-positive, CD45-negative cells with an intact nucleus (DAPI+) that is positioned at least 50% within the cytoplasm, and has a diameter of at least 4×4 μm were defined as CTCs. Each sample was analysed independently by two readers and questionable events evaluated until consensus was reached. The results were reported as number of CTCs/7.5ml whole blood (Figure 1).

To determine whether the platform was capable of detecting cells from HNSCC spiking experiments using the epithelial FaDu cell line (ATCC^®^HTB43^TM^) and CAL27 (ATCC^®^CRL2095^TM^) was performed (Supplementary Figure 2 and 3). As a control, 4 further healthy volunteers were screened and no CTC-events were observed.

### Enrichment of circulating tumour cells using ScreenCell^®^

Whole blood (6 ml) was collected in EDTA vacutainers and processed within 4 hours. ScreenCell^®^ Cyto (ScreenCell^®^, France) microfiltration devices were used to capture CTCs according to the manufacturers protocol. Briefly, 3 ml of peripheral blood was mixed with 4 ml of ScreenCell^®^ FC2 dilution buffer. The content was homogenised by inverting 5 times followed by an incubation of 8 minutes at room temperature. The 7 ml of diluted blood was then filtered through the ScreenCell^®^ Cyto (ScreenCel^®^l, France) (filter pore size 7.5μm) device by using vacuum force provided by a 9 ml vacutainer. This step was followed by rinsing the filter with 1.6 ml of 1xPBS. The circular filter was then dried on whatman paper and stained. Initial staining was with H&E (Harris) followed by immunofluorescent staining for cytokeratins 8/18/19, CD45 (CellSearch^®^ antibody mixture) (1:6 dilution), EGFR (Biolegend, AY13) and DAPI (Figure 2). Imaging performed using an Olympus IX3 inverted microscope. Furthermore, to verify whether the platform was capable of detecting cells from HNSCC, spike-in experiments were conducted with the FaDu cell line (ATCC^®^HTB43^TM^) (Supplementary Figure 4). As a control, 4 healthy volunteers were screened and no CTC-events were observed.

### Enrichment of circulating tumour cells using RosetteSep^®^

Whole blood collected in lithium heparin tubes (StemCell Technologies, Vancouver, Canada) was incubated with RosetteSep^®^ Human CD45 depletion cocktail (Stemcell Technologies, Vancouver, British Columbia, Canada) at 50 μl/mL of whole blood and incubated for 20 minutes at room temperature. Then CTC separation is made in a density gradient using SepMate™-50 mL tubes containing Lymphoprep™ (Stemcell Technologies, Vancouver, Canada). For microscopy, the enriched cells were cytospun (1000 rpm, 5mins) onto slides and stained with a combination of antibodies to cytokeratins 8/18/19, CD45 (CellSearch^®^ antibody mixture) (1:6 dilution), EGFR (Biolegend, AY13) and DAPI and analysed using an Olympus IX3 inverted microscope (Figure 3). Furthermore, to verify whether this method was capable of detecting cells from HNSCC, spike-in experiments were conducted with the FaDu cell line (ATCC^®^HTB43^TM^) (Supplementary Figure 5). To determine the viability of the cells post enrichment, the spike-in tumour cell fraction was put into standard media (RPMI-1640 Glutamax), supplemented with 10% foetal bovine serum and 1% Penicillin-Streptomycin and incubated (Life Technologies, Inc) at 37°C and 5% CO_2_.

### Detection of CTCs

CTCs were visualized using immunofluorescence on the ScreenCell^®^ filters and RosetteSep^TM^ enriched cytospins using a number of markers. We classified cells as CTCs if they were positive for EGFR, cytokeratins 8/18/19, negative for CD45, had intact nuclei and morphologically different from leucocytes. CTCs tended to be larger than the surrounding leukocytes, however this should not be used as a general rule as CTCs have been shown to vary in size.

## SUPPLEMENTARY MATERIALS FIGURES AND TABLES



## References

[1] Schmidt H, Kulasinghe A, Perry C, Nelson C, Punyadeera C (2016). A liquid biopsy for head and neck cancers. Expert review of molecular diagnostics.

[2] He S, Li P, He S, Long T, Zhang N, Fang J, Yu Z (2013). Detection of circulating tumour cells with the CellSearch system in patients with advanced-stage head and neck cancer: preliminary results. The Journal of laryngology and otology.

[3] Jatana KR, Teknos TN, Chalmers JJ, Balasubramanian P, Lang JC, Yang LY, Jatana CA, White E, Agrawal A, Ozer E, Schuller DE (2010). Significance of Circulating Tumor Cells in Patients With Squamous Cell Carcinoma of the Head and Neck. ARCHIVES OF OTOLARYNGOLOGY-HEAD & NECK SURGERY.

[4] Trosman SJ, Koyfman SA, Ward MC, Al-Khudari S, Nwizu T, Greskovich JF, Lamarre ED, Scharpf J, Khan MJ, Lorenz RR, Adelstein DJ, Burkey BB (2015). Effect of human papillomavirus on patterns of distant metastatic failure in oropharyngeal squamous cell carcinoma treated with chemoradiotherapy. JAMA otolaryngology-- head & neck surgery.

[5] O'sullivan B, Huang SH, Su J, Garden AS, Sturgis EM, Dahlstrom K, Lee N, Riaz N, Pei X, Koyfman SA, Adelstein D, Burkey BB, Friborg J (2016). Development and validation of a staging system for HPV-related oropharyngeal cancer by the International Collaboration on Oropharyngeal cancer Network for Staging (ICON-S): a multicentre cohort study. The Lancet Oncology.

[6] Chai RC, Lambie D, Verma M, Punyadeera C (2015). Current trends in the etiology and diagnosis of HPV-related head and neck cancers. Cancer medicine.

[7] Huang SH, Xu W, Waldron J, Siu L, Shen X, Tong L, Ringash J, Bayley A, Kim J, Hope A, Cho J, Giuliani M, Hansen A (2015). Refining American Joint Committee on Cancer/Union for International Cancer Control TNM stage and prognostic groups for human papillomavirus-related oropharyngeal carcinomas. Journal of clinical oncology.

[8] Ang KK, Harris J, Wheeler R, Weber R, Rosenthal DI, Nguyen-Tan PF, Westra WH, Chung CH, Jordan RC, Lu C, Kim H, Axelrod R, Silverman CC (2010). Human papillomavirus and survival of patients with oropharyngeal cancer. The New England journal of medicine.

[9] O'sullivan B, Huang SH, Perez-Ordonez B, Massey C, Siu LL, Weinreb I, Hope A, Kim J, Bayley AJ, Cummings B, Ringash J, Dawson LA, Cho BC (2012). Outcomes of HPV-related oropharyngeal cancer patients treated by radiotherapy alone using altered fractionation. Radiotherapy and oncology.

[10] Huang SH, Perez-Ordonez B, Liu FF, Waldron J, Ringash J, Irish J, Cummings B, Siu LL, Kim J, Weinreb I, Hope A, Gullane P, Brown D (2012). Atypical clinical behavior of p16-confirmed HPV-related oropharyngeal squamous cell carcinoma treated with radical radiotherapy. International journal of radiation oncology, biology, physics.

[11] Misiukiewicz K, Camille N, Gupta V, Bakst R, Teng M, Miles B, Genden E, Sikora A, Posner M (2014). The role of HPV status in recurrent/metastatic squamous cell carcinoma of the head and neck. Clinical advances in hematology & oncology.

[12] Kulasinghe A, Perry C, Jovanovic L, Nelson C, Punyadeera C (2015). Circulating tumour cells in metastatic head and neck cancers. International journal of cancer.

[13] Hillig T, Horn P, Nygaard AB, Haugaard AS, Nejlund S, Brandslund I, Soletormos G (2015). In vitro detection of circulating tumor cells compared by the CytoTrack and CellSearch methods. Tumour biology.

[14] Khoo BL, Lee SC, Kumar P, Tan TZ, Warkiani ME, Ow SG, Nandi S, Lim CT, Thiery JP (2015). Short-term expansion of breast circulating cancer cells predicts response to anti-cancer therapy. Oncotarget.

[15] Aceto N, Bardia A, Miyamoto DT, Donaldson MC, Wittner BS, Spencer JA, Yu M, Pely A, Engstrom A, Zhu H, Brannigan BW, Kapur R, Scott SL (2014). Circulating tumor cell clusters are oligoclonal precursors of breast cancer metastasis. Cell.

[16] McInnes LM, Jacobson N, Redfern A, Dowling A, Thompson EW, Saunders CM (2015). Clinical implications of circulating tumor cells of breast cancer patients: role of epithelial-mesenchymal plasticity. Frontiers in oncology.

[17] Pantel K, Alix-Panabieres C (2013). Real-time liquid biopsy in cancer patients: fact or fiction?. Cancer research.

[18] Alix-Panabieres C, Pantel K (2013). Circulating tumor cells: liquid biopsy of cancer. Clinical chemistry.

[19] Bozec A, Ilie M, Dassonville O, Long E, Poissonnet G, Santini J, Chamorey E, Ettaiche M, Chauviere D, Peyrade F, Hebert C, Benezery K, Sudaka A (2013). Significance of circulating tumor cell detection using the CellSearch system in patients with locally advanced head and neck squamous cell carcinoma. European archives of oto-rhino-laryngology.

[20] Duray A, Lacremans D, Demoulin S, Delvenne P, Saussez S (2014). Prognosis of HPV-positive head and neck cancers: implication of smoking and immunosuppression. Advances in Cellular and Molecular Otolaryngology.

[21] Chai RC, Lim Y, Frazer IH, Wan Y, Perry C, Jones L, Lambie D, Punyadeera C (2016). A pilot study to compare the detection of HPV-16 biomarkers in salivary oral rinses with tumour p16(INK4a) expression in head and neck squamous cell carcinoma patients. BMC cancer.

[22] Allard WJ, Matera J, Miller MC, Repollet M, Connelly MC, Rao C, Tibbe AG, Uhr JW, Terstappen LW (2004). Tumor cells circulate in the peripheral blood of all major carcinomas but not in healthy subjects or patients with nonmalignant diseases. Clinical cancer research.

[23] Gao D, Vela I, Sboner A, Iaquinta PJ, Karthaus WR, Gopalan A, Dowling C, Wanjala JN, Undvall EA, Arora VK, Wongvipat J, Kossai M, Ramazanoglu S (2014). Organoid cultures derived from patients with advanced prostate cancer. Cell.

[24] Drost J, Karthaus WR, Gao D, Driehuis E, Sawyers CL, Chen Y, Clevers H (2016). Organoid culture systems for prostate epithelial and cancer tissue. Nature protocols.

[25] Gasch C, Plummer PN, Jovanovic L, McInnes LM, Wescott D, Saunders CM, Schneeweiss A, Wallwiener M, Nelson C, Spring KJ, Riethdorf S, Thompson EW, Pantel K (2015). Heterogeneity of miR-10b expression in circulating tumor cells. Scientific reports.

[26] Kulasinghe A, Perry C, Warkiani ME, Blick T, Davies A, O'Byrne K, Thompson EW, Nelson CC, Vela I, Punyadeera C (2016). Short term ex-vivo expansion of circulating head and neck tumour cells. Oncotarget.

[27] Hristozova T, Konschak R, Stromberger C, Fusi A, Liu Z, Weichert W, Stenzinger A, Budach V, Keilholz U, Tinhofer I (2011). The presence of circulating tumor cells (CTCs) correlates with lymph node metastasis in nonresectable squamous cell carcinoma of the head and neck region (SCCHN). Annals of oncology.

[28] Yang L, Lang JC, Balasubramanian P, Jatana KR, Schuller D, Agrawal A, Zborowski M, Chalmers JJ (2009). Optimization of an enrichment process for circulating tumor cells from the blood of head and neck cancer patients through depletion of normal cells. Biotechnology and bioengineering.

[29] Hristozova T, Konschak R, Budach V, Tinhofer I (2012). A simple multicolor flow cytometry protocol for detection and molecular characterization of circulating tumor cells in epithelial cancers. Cytometry Part A.

[30] Balasubramanian P, Yang L, Lang JC, Jatana KR, Schuller D, Agrawal A, Zborowski M, Chalmers JJ (2009). Confocal images of circulating tumor cells obtained using a methodology and technology that removes normal cells. Molecular pharmaceutics.

[31] Stoecklein NH, Fischer JC, Niederacher D, Terstappen LW (2016). Challenges for CTC-based liquid biopsies: low CTC frequency and diagnostic leukapheresis as a potential solution. Expert review of molecular diagnostics.

[32] Hendrix MJ, Seftor EA, Seftor RE, Chao JT, Chien DS, Chu YW (2016). Tumor cell vascular mimicry: Novel targeting opportunity in melanoma. Pharmacology & therapeutics.

[33] Desitter I, Guerrouahen BS, Benali-Furet N, Wechsler J, Janne PA, Kuang Y, Yanagita M, Wang L, Berkowitz JA, Distel RJ, Cayre YE (2011). A new device for rapid isolation by size and characterization of rare circulating tumor cells. Anticancer research.

[34] Kulemann B, Pitman MB, Liss AS, Valsangkar N, Fernandez-Del Castillo C, Lillemoe KD, Hoeppner J, Mino-Kenudson M, Warshaw AL, Thayer SP (2015). Circulating tumor cells found in patients with localized and advanced pancreatic cancer. Pancreas.

[35] Fina E, Reduzzi C, Motta R, Di Cosimo S, Bianchi G, Martinetti A, Wechsler J, Cappelletti V, Daidone MG (2015). Did circulating tumor cells tell us all they could? The missed circulating tumor cell message in breast cancer. The International journal of biological markers.

[36] Buglione M, Grisanti S, Almici C, Mangoni M, Polli C, Consoli F, Verardi R, Costa L, Paiar F, Pasinetti N, Bolzoni A, Marini M, Simoncini C (2012). Circulating tumour cells in locally advanced head and neck cancer: preliminary report about their possible role in predicting response to non-surgical treatment and survival. European journal of cancer (Oxford, England : 1990).

[37] Nichols AC, Lowes LE, Szeto CC, Basmaji J, Dhaliwal S, Chapeskie C, Todorovic B, Read N, Venkatesan V, Hammond A, Palma DA, Winquist E, Ernst S (2012). Detection of circulating tumor cells in advanced head and neck cancer using the CellSearch system. Head & neck.

[38] Xu L, Mao X, Imrali A, Syed F, Mutsvangwa K, Berney D, Cathcart P, Hines J, Shamash J, Lu YJ (2015). Optimization and Evaluation of a Novel Size Based Circulating Tumor Cell Isolation System. PloS one.

[39] Janssen Diagnostics LLC (2016). CTCs Above or Below a Predetermined Cutoff Number Predicit Prognosis.

[40] Warkiani ME, Khoo BL, Wu L, Tay AK, Bhagat AA, Han J, Lim CT (2016). Ultra-fast, label-free isolation of circulating tumor cells from blood using spiral microfluidics. Nature protocols.

[41] Ramirez JM, Fehm T, Orsini M, Cayrefourcq L, Maudelonde T, Pantel K, Alix-Panabieres C (2014). Prognostic relevance of viable circulating tumor cells detected by EPISPOT in metastatic breast cancer patients. Clinical chemistry.

