# Supplementary material for: Impact of label-free technologies in head and neck cancer circulating tumour cells
Source: Oncotarget. 2016 Sep 16;7(44):71223–34. doi: 10.18632/oncotarget.12086 (PMC5342074; doi:10.18632/oncotarget.12086)
Supplement: Supplementary file 1 [file oncotarget-07-71223-s001.pdf]

Impact of label-free technologies in head and neck cancer circulating tumour cells

Supplementary Material

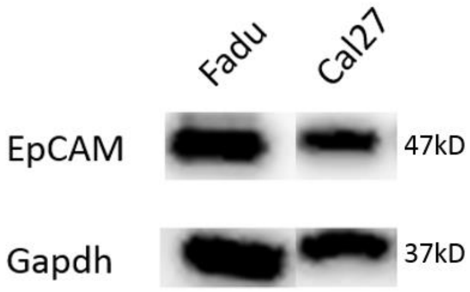

Supplementary figure 1. Western blot of HNSCC cell lines FaDu and Cal27 positive for EpCAM

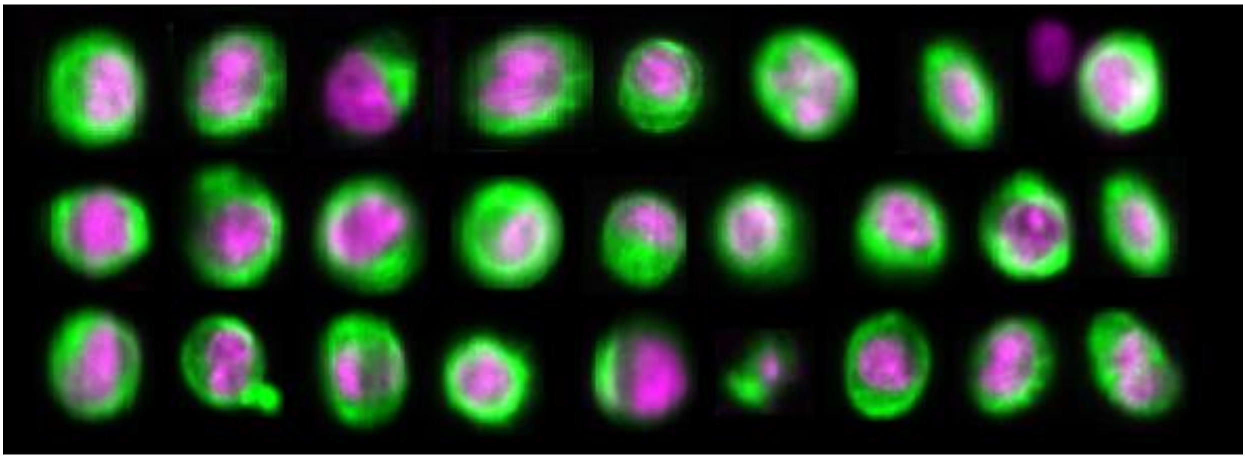

Supplementary figure 2. Presentation of spiked-in and recovered tumour cells [FaDu (ATCC®HTB43™, Cal27 (ATCC®CRL2095™)] into 7.5ml of normal healthy volunteer blood collected in CellSave tubes. Image gallery presenting tumour cells detected using the CellSearch technology: EpCAM+Cytokeratin+Dapi+CD45- with a diameter of at least 4μm. The regular morphology (large and round) of the 2 cell lines is evident in contrast to the patient CTCs. Imaged on the Celltracks Analyzer II ® system.

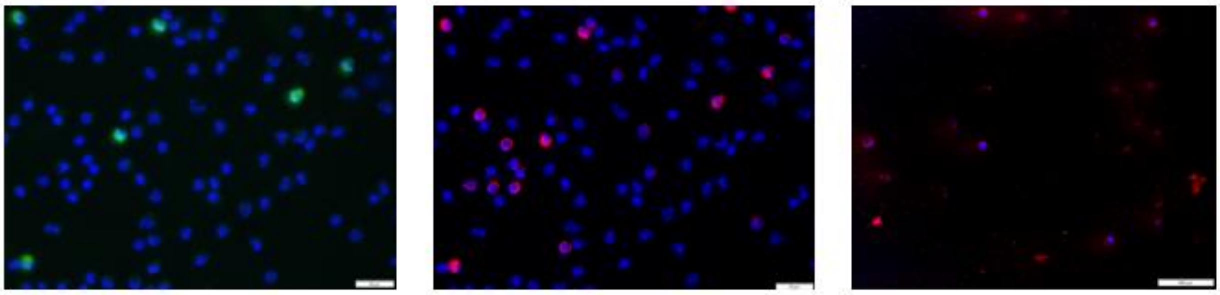

Supplementary figure 3. Presentation of spiked-in and recovered tumour cells FaDu (ATCC®HTB43™) into 7.5 ml of normal healthy volunteer blood collected in EDTA tubes. Image presenting tumour cells detected using the ScreenCell technology: Cytokeratin+Dapi+CD45- with a diameter of at least 7µm. Scale bars represent 50 µm.

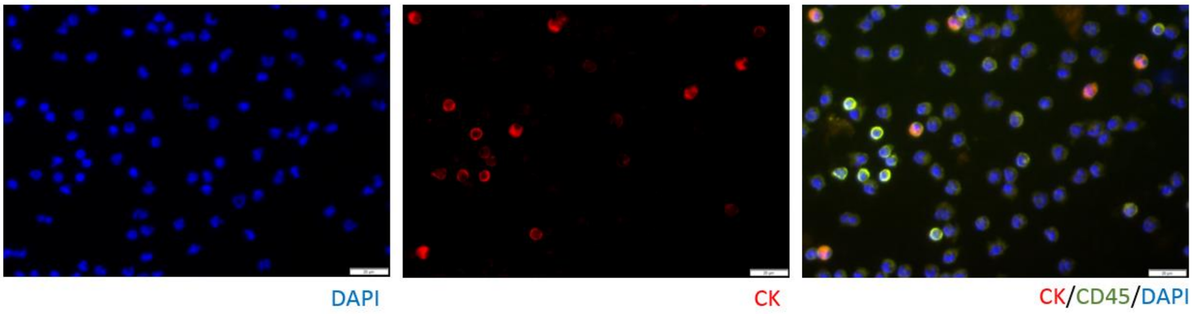

Supplementary figure 4. Presentation of spiked-in and recovered tumour cells FaDu (ATCC®HTB43™) into 7.5 ml of normal healthy volunteer blood collected in Heparinized tubes. Image presenting tumour cells enriched using the RosetteSep technology: Cytokeratin+Dapi+CD45-. Remaining Leukocytes post enrichment are Dapi+CD45+.

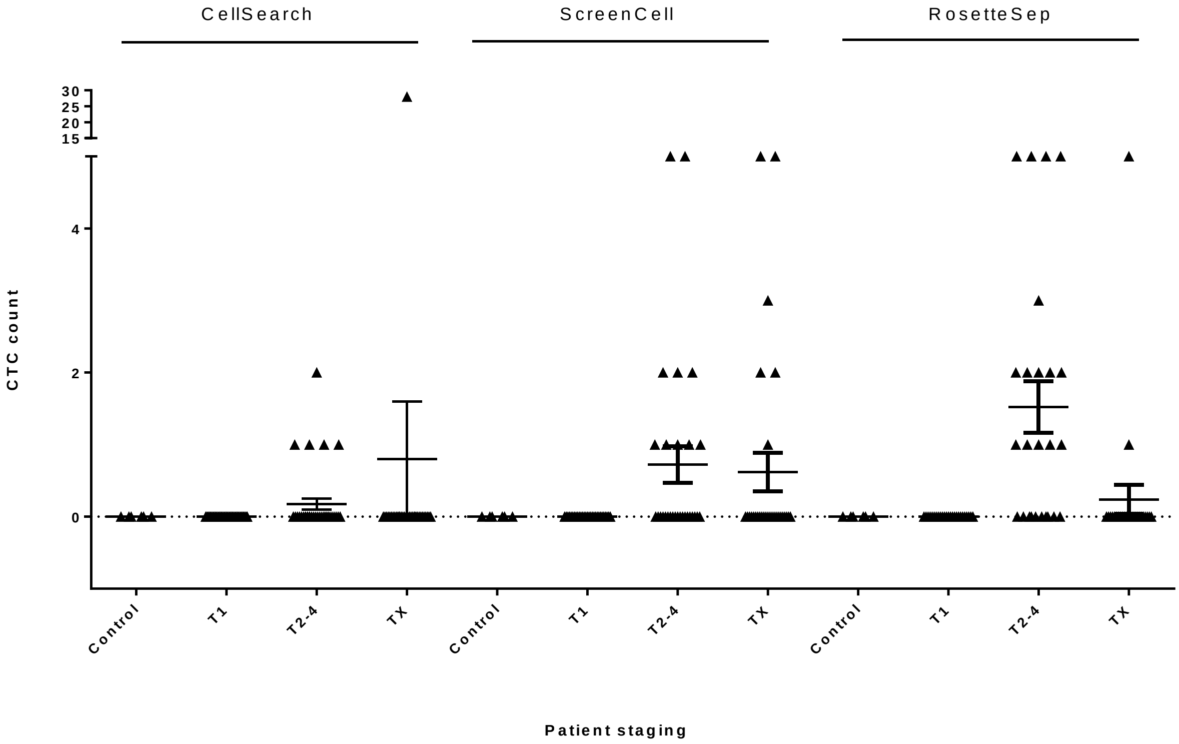

Supplementary figure 5. Figure representing Tumour staging (T) with CTC detection across the 3 CTC enrichment platforms. Higher T staging correlated with a higher CTC detection. Control samples were from normal healthy volunteers.

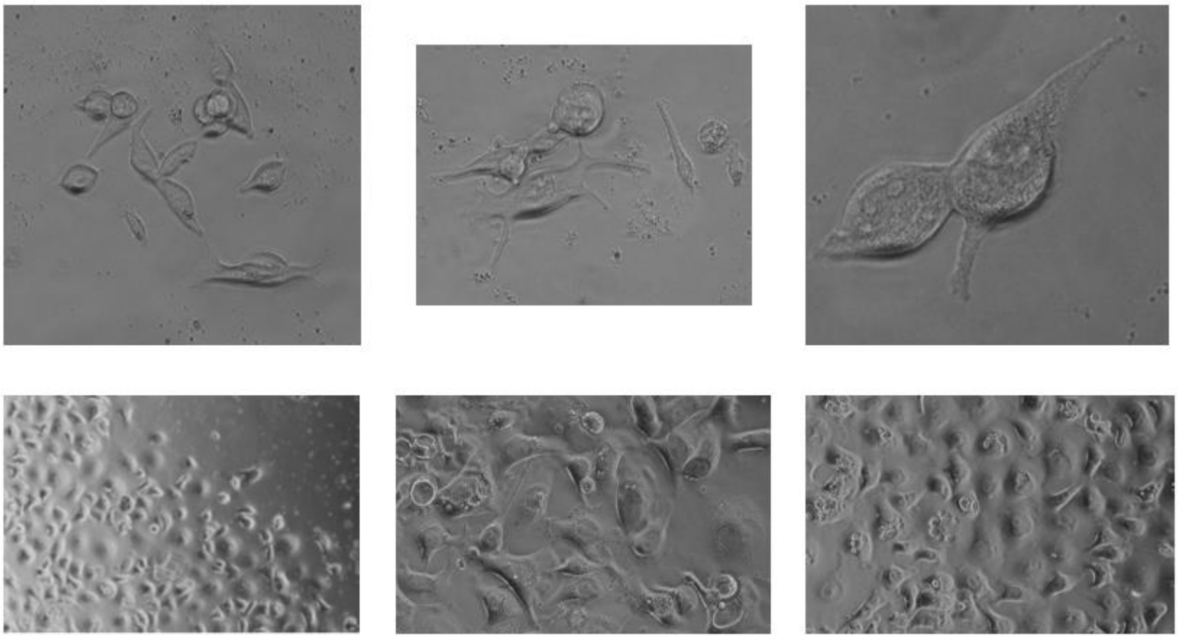

Supplementary figure 6. Presentation of spiked-in and recovered tumour cells FaDu (ATCC®HTB43™) into 7.5 ml of normal healthy volunteer blood collected in Heparinized tubes. Image presenting tumour cells enriched using the RosetteSep technology and put into standard culture to determine the viability of the cells post enrichment. The first row shows cells at day 5 and the second row shows cells at day 10.

Supplementary table 1. Head to head comparison (CellSearch vs ScreenCell n=27)

TNM Staging		CellSearch		ScreenCell
TxN2bM0		-		2 CTCs
T4N0M0		-		2 Clusters
T4N2bM0		-		-
TxN0M0		28 CTCs		3 CTCs
T3N0M0		-		-
T4N2cM1		-		2 CTCs
TxN2bM0		-		1 CTC
T3N0M0		-		1 Cluster
TxN2bM0		-		2 CTCs + Cluster
T4aN2bM0		1 CTC		2 CTCs
T4N1M0		-		1 CTC
TxN2bM0		-		2 CTCs + Cluster
T4aN0M0		-		filter blocked
T4aN2bM0		-		CTC cluster
T2N0M0		-		2 CTCs
T4N2bM0		-		1 CTC
T2N2bM0		-		-
T4N2cM0		1 CTC		-
T3N2bM0		2 CTCs		-
T4aN2bM0		1 CTC		-
T2N2aM0		-		-
T4N2CM0		-		-
T2N0M0		-		-
T4N2bM0		2 CTCs		-
T4N2CM0		-		-
T4N0M0		-		-
T4aN2CM0		-		-

Supplementary table 2. Head to head comparison (CellSearch vs RosetteSep n=10)

TNM Staging		CellSearch		RosetteSep
TxN2bM0				CTC Cluster
T4N0M0		1 CTC		1 CTC
T3N2aM0				1 CTC
T4aN2bM0				1 CTC
T4N2cM0				
T4N2cM0				
T4BN2bM0				CTC cluster
T4aN2cM0				
T4aN2aM0				CTC+clusters
T4aN2aM0				
